# Prevalence of HIV infection and uptake of HIV/AIDS services among fisherfolk in landing Islands of Lake Victoria, north western Tanzania

**DOI:** 10.1186/s12913-018-3784-4

**Published:** 2018-12-18

**Authors:** Anthony Kapesa, Namanya Basinda, Elias C. Nyanza, Martha F. Mushi, Ola Jahanpour, Sospatro E. Ngallaba

**Affiliations:** 1 Department of Community Medicine, School of Public Health, Catholic University of Health and Allied Sciences (CUHAS), P.O. BOX 1464, Mwanza, Tanzania; 2 Department of Environmental and occupational Health & Geographical information system, School of Public Health, Catholic University of Health and Allied Sciences, Mwanza, Tanzania; 3 Department of Microbiology and Immunology, School of Medicine, Catholic University of Health and Allied Sciences, Mwanza, Tanzania; 4 Institute of Public Health, Department of Epidemiology and Biostatistics, Kilimanjaro Christian Medical University College, Moshi, Tanzania

**Keywords:** HIV infection, Fisherfolk, Landing islands, HIV/AIDS services, Tanzania

## Abstract

**Background:**

New HIV infections in Tanzania have been decreasing, however some populations remain at higher risk. Despite of that, evidence on the magnitude of HIV infection and the associated factors and HIV/AIDS services uptake among fisherfolk in Tanzania are inadequately explored. This study therefore aimed at determining prevalence of HIV infection and utilization of HIV/AIDS services among fishfolk in selected Islands of Lake Victoria for evidence-based interventions.

**Methods:**

Cross-sectional study determining status of HIV infection among fisherfolk (*n* = 456) and retrospective review of voluntary counselling and testing (VCT) registry (*n* = 1744) were done in Buchosa and Muleba districts. Structured questionnaire and HIV rapid test kits with the standard testing protocol were used as research tools.

**Results:**

A total of 269 (58.9%) male and 187 (41.1%) female fisherfolk were recruited during the community survey. Prevalence of HIV infection was 14% in all surveyed landing sites with a site variation from as low as 7.2% to as high as 23.8%. Participants employed in fishing related employment had higher odds of being HIV infected (5.4 times) than those who practiced fishing and partly farming [OR = 5.40; 95%CI 1.88–15.61; *p* < 0.001]. Participants employed in fishing related employment had higher odds of being HIV infected (5.4 times) than those practiced fishing and farming [OR = 5.40; 95%CI 1.88–15.61; *P* < 0.001]. Lack of formal education [aOR = 3.37; 95%CI 1.64–6.92; *p* < 0.001], being older [aOR = 1.06; 95%CI 1.03–1.09] and using alcohol [aOR = 2.26; 95%CI 1.23–4.15] predicted the likelihood of contracting HIV infection. Approximately three quarters (76%) of respondents had ever tested for HIV infection within past 1 year. Moreover, about half of the study participants had used condom inconsistently and 5 out of 14 (37.5%) of participants who knew their status had never started treatment. Despite the low uptake of most HIV preventive services, majority (88%) of male fisherfolk were circumcised.

**Conclusion:**

The magnitude of HIV infection among fisherfolk was up to 3 times higher than that of the general populations in Muleba and Buchosa districts. Higher age, using alcohol and lack of formal education predicted increased likelihood of HIV infection. The uptake of key HIV/AIDS curative and preventive services was generally low.

**Electronic supplementary material:**

The online version of this article (10.1186/s12913-018-3784-4) contains supplementary material, which is available to authorized users.

## Background

HIV pandemic is the major public health challenge of the present decade with about 36.7 million people lived with HIV/AIDS globally in the year 2015 with only 17 million on antiretroviral treatment (ART) [1]. Worldwide, it is estimated that 70% of the HIV-infected individuals are living in Sub-Saharan Africa [2]. HIV/AIDS is responsible for social and economic growth impediment in developing countries affecting all sectors together without excluding fishing communities [3].

New HIV infections in Tanzania have been decreasing [4] however some populations are still at higher risk than the general population [5]. These key and vulnerable populations include; commercial sex workers, intra venous (IV) drug abusers and mobile population like, fishermen, prisoners and truck drivers [6–9]. In India, fishermen were reported to have uppermost HIV infection vulnerability however similar observation in Kenya and Uganda has been reported [10–13]. Factors contributed to such high infection transmission among other factors were frequent mobility and excessive alcohol use before sexual intercourse [10, 14, 15].

High mobility and limited number of care and treatment facilities among fisherfolk has also being reported as the main challenge facing utilization of ART services [10]. Access to antiretroviral treatment in Tanzania has been improving but the status in hard to reach areas like fishing islands of Lake Victoria remains not well explored despite of it being mentioned as a challenge among fisherfolk elsewhere in Africa [16]. In Tanzania, fisheries sector provides income, foreign export revenue and most importantly responsible for over 4 million jobs with about 2.4% of the country’s Gross Domestic Product [17, 18]. As fisheries become more unified into the National economy and employment of many stakeholders, the chances that mobile fisherfolk become a bridge population linking areas with high HIV transmission and those with low magnifies. Therefore, the impact of HIV/AIDs among fisherfolk may seriously affect countries’ economic growth and expansion. This study then aimed at understanding the magnitude of HIV infection and uptake of HIV/AIDS services in hard to reach fisherfolk landing sites in Lake Victoria where there is a limited data.

## Methods

### Study settings

**Fig. 1:**
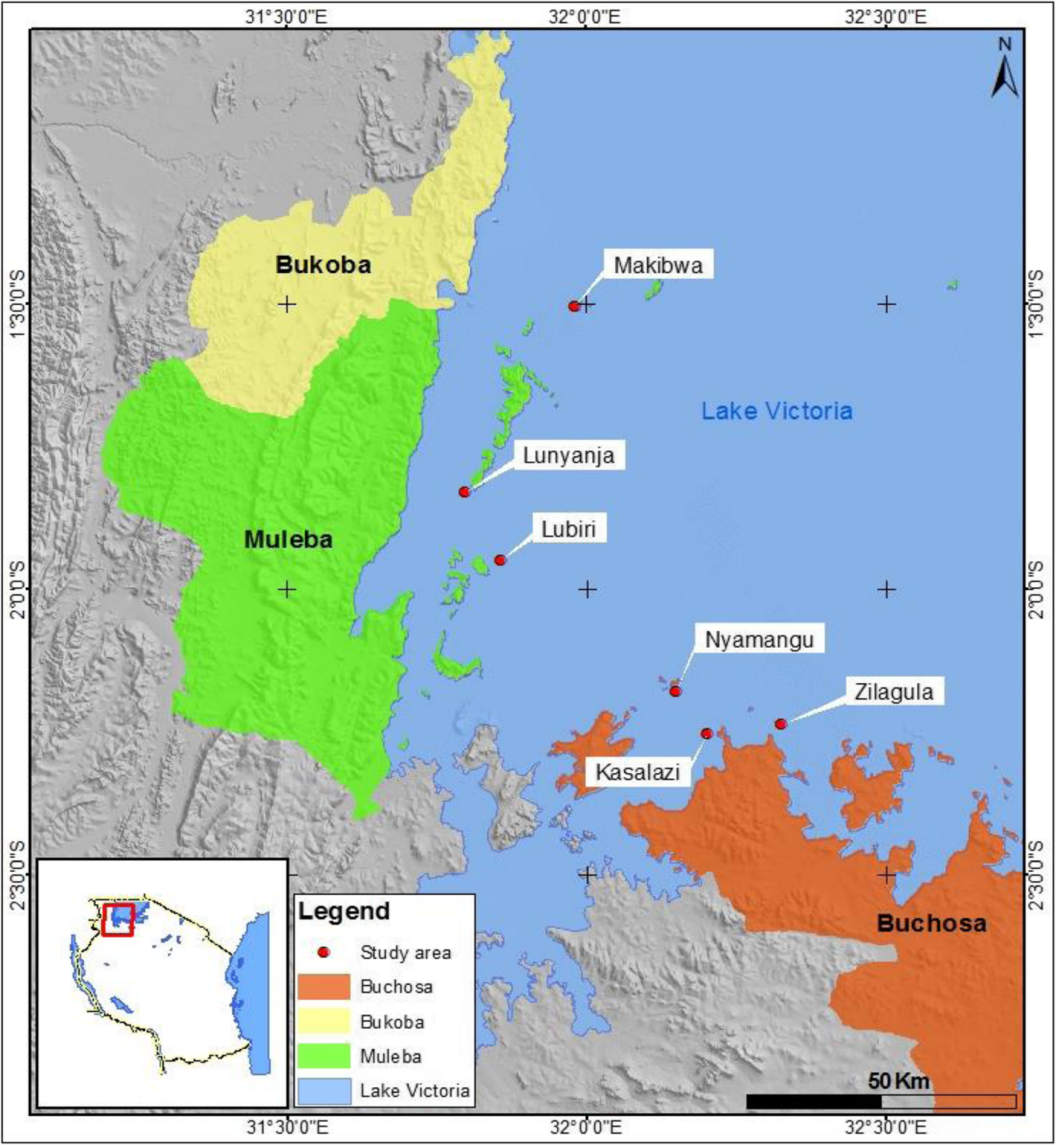
Location of the study area showing the six surveyed fisherfolk landing sites located in Muleba and Buchosa Districts, North western Tanzania

This study was conducted in 13 fisherfolk landing islands located in Buchosa District (Mwanza region) and Muleba District (Kagera region) as described by Fig. 1. Three hard to reach fisherfolk landing sites (fishing camps with at least 500 fisherfolk) from each District were conveniently selected during the community based survey. Moreover, retrospective review of VCT registry was conducted in ten landing sites located within localities the surveyed sites. Muleba District is one of the 7 Districts in Kagera Region. The Region has an estimated population of 1,849,965. Muleba District has an area of 10,739 km^2^ whereby 76.8% the area is part of Lake Victoria. Kagera Region has a total of 143 landing sites with approximately 21,700 fisherfolk but only 23% of the sites are being provided with ART services [19]. On the other hand, Mwanza Region has 8 Districts with an estimated population of 3,500,000 in which Buchosa District is part. Buchosa District has an area of 6657 square km whereby 81.9% the area is part of Lake Victoria, the district has a population of 327,767. Mwanza Region has a total of 243 landing sites with a total of 45,616 fisherfolk, only 31% of sites are provided with ART services [19]. Mwanza and Kagera constitute two of three regions in Tanzania that have shown HIV transmission upsurge recently [20]. Moreover, the selected study sites had no direct access to health services, mobile clinics including that of the Jubilee Hope medical ship served the sites collaboratively with the District Health authorities. Additionally, the landing sites/fishing camps are mainly made of temporally structures used as bars, apartments and guest houses.

### Study design and study population

A cross-sectional study among fisherfolk placed in landing islands was done in Buchosa and Muleba between September and October 2017. The survey involved 456 fisherfolk who voluntarily agreed to participate in the study. Kish Leslie formula [21] for cross-sectional surveys was used to estimate the study sample size basing on the previously established prevalence in neighbouring countries [10, 13]. Additional retrospective cross-sectional study reviewing the VCT registry collected by Jubilee Hope mobile clinic within or near the study areas was done. Results for clients who voluntarily tested for HIV during the Jubilee Hope Mobile Clinic visits in Muleba and Buchosa between September and October in 2017 were entered in the CTC registry as required by the National HIV/AIDS control program. A total of 1744 fisherfolk who voluntarily tested for HIV were included in the study.

### Sampling and participants recruitment

A list of residential and business huts was obtained from local leadership among the conveniently selected islands, a systematic random selection of 323 huts in all surveyed island out of 1922 huts was done. The selected huts were visited one after the other, all individuals above the age of 15 found in the hut were asked to participate. On the other hand, review of CTC registry conveniently involved clients who were tested 2 months prior the community survey.

### Data collection

This study used a pre-tested structured questionnaire to explore social demographics, HIV/AIDS knowledge, attitude, and uptake of HIV/AIDS services. Before administration the questionnaire all respondents were asked to participate in the study before signing an informed consent form. Knowledge of HIV/AIDS among fisherfolk was assessed by using 30 items which included questions regarding disease presentation, transmission of HIV/AIDS and preventive measures (Additional file 1). Responses on knowledge assessments were assigned as correct response, not sure and wrong response. Attitude towards HIV/AIDS was assessed by 15 positive and negative statements covering the cause, infection susceptibility and transmission as well as prevention (Additional file 1). Responses on attitude adopted a simple Likert scale with agree, disagree and not sure options. Determination of uptake of HIV/AIDS services encompassed condom use for past 12-month, HIV test for 12-month, ARV use and male circumcision. Diagnosis of HIV infection was done using rapid HIV testing algorithm as recommended by the Ministry of Health, Alere (US) SD Bioline® started followed by Alere (US) Determine® before Trinity Biotech (Ireland) Uni-Gold® for confirmation [22]. Counselling before and after testing was thoroughly done while observing sterility condition during pricking.

During review of VCT registry, information abstracted included; age, sex, residence and test results. Diagnosis of HIV infection during VCT delivered by the mobile clinic adhered to the Tanzanian HIV rapid test algorithm [23].

### Data management and analysis

Data were entered in excel® window 10 and cross-checked for errors before being exporting and analysing by Statistical Software for Social Sciences (SPSS® Version 17). Level of HIV knowledge was considered appropriate when the respondents scored more than 20 points whilst less or equal to 20 was assigned as inappropriate. Measurement of knowledge was based on 30 items with three options (correct/agree, not correct/disagree and not sure/do not know with 1, 0.5 and 0 scores respectively). Positive attitude was considered when the respondent scored more than 8 points while negative when the score less or equal to 8. Simple Likert scale was used to assess attitude; agree, not sure and disagree were used with scores of 0, 0.5 and 1 respectively. Chi-square test, confidence interval and *p* < 0.05 were used to measure the association. Student t-test and Mann Whitney rank sum test were also employed where appropriate. Unadjusted and an adjusted logistic regression analysis were done. Variable with a *p* value < 0.2 at an unadjusted logistic regression analysis, were entered in an adjusted mode using forward stepwise to select the variables in the final model. A p value of < 0.05 was considered as statistically significant. Hosmer-Lemeshow test and overall prediction percentage were used to select the best model.

## Results

### Social demographics of the study participants

A total of 456 study participants were recruited in this study with more men than women (58.9% vs 41.1%). Mean age of participants was 32.97 ± 9.68 years ranging from 15 to 80 years. Majority of the study participants (43.9%) were fishermen and minority (15.1%) being employed in different fishing related activities (fish drying, cooking and store keeping). Three-quarters of the respondents were mobile population conducting fish related activities and the rest as permanent residents fully or partly engaged in fishing. The distribution of residence in terms of being permanent or mobile was comparable between the two studied Districts [χ^2^ = 5.89; df = 1; *p* > 0.05]. Table 1 shows the details the social demographics.

**Table 1:** Social demographic attributes of the study participants in fishing landing sites of Muleba and Buchosa Districts, September to October 2017, north-western Tanzania

Variable	Respondents location		*p*-value
	Muleba (*N* = 254) (%)	Buchosa (*N* = 202) (%)	
Mean age (in years)	33.01	32.92	> 0.05
Sex			
Men	169 (67)	100 (50)	
Women	85 (33)	102 (50)	< 0.001
Marital status			
Married	157(62)	102 (50)	
Single	65 (26)	40 (20)	< 0.001
Divorced/Widow/Cohabit	32 (13)	60 (30)	
Education			
No formal education	22 (9)	32 (16)	
Primary+	232 (91)	170 (84)	> 0.05
Occupation			
Fishing	128 (50)	72 (35)	
Fishing related business	57 (22)	38 (19)	
Fishing related employment	23 (10)	46 (23)	> 0.05
Farming and fishing	46 (18)	46 (23)	
Religion			
Christian	206 (81)	177 (88)	
Muslim	48 (19)	25 (12)	> 0.05
Residence			
Permanent	77 (30)	41 (20)	
Only for business	177 (70)	161 (80)	> 0.05

### Prevalence HIV infection during the community-based survey

This study found 14% prevalence of HIV infection among fisherfolk with a variation from as low as 7.2% at Lunyanja to as high as 23.8% at Kasalazi (Table 2). HIV prevalence was higher among fisherfolk located in Buchosa District compared to those from Muleba District (18% vs 10.9%). The odds of having HIV infection was higher in Buchosa District than in Muleba District [OR = 1.79; 95%CI 1.04–3.06; *p* < 0.05]. The infection was lower among men than women (12% vs 17.1%) and yet statistically not different [OR = 0.662; 95% CI 0.388–1.129; *p* > 0.05]. In Buchosa District, the odds of contracting HIV among men was lower compared to women [OR = 0.432; 95%CI 0.202–0.921; *p* < 0.05] whilst comparable in Muleba District [OR = 1.448; 95%CI 0.586–3.579; *p* > 0.05]. Prevalence of HIV infection was highest (29.3%) among the combination of the widowed, divorced and cohabiting participants and lowest (9.5%) among the married respondents. Participants employed in fish related employment had higher odds of being HIV infected (5.4 times) than those who practiced fishing and partly farming [OR = 5.40; 95%CI 1.88–15.61; *p* < 0.001]. During the survey however, all respondents accepted HIV testing with exception of only 2%.

**Table 2:** Prevalence of HIV infection among fisherfolk in some landing islands of Muleba and Buchosa District during a community survey in October 2017 (*N* = 447)

Island	HIV test results		Total tested	Prevalence (%)
	Positive	Negative		
Lubiri	8	72	80	10
Makibwa	13	71	84	18
Nyamangu	8	69	77	11.4
Kasalazi	15	48	63	23.8
Lunyanja	6	77	83	7.2
Zilagula	13	47	60	21.7
Overall	63	384	447	14

### Magnitude of HIV sero-positivity from the Voluntary Counselling and Testing (VCT) Registry

Prevalence of HIV infection during VCT program among 1744 clients was 9.1%. Odds of HIV infection between the two Districts was comparable [OR = 1.250; CI 95% 0.812–1.925; *p* > 0.05]. However, lowers odds of HIV infection among men than women was observed in Muleba District [OR = 0.414; 95%CI 0.286–0.600, *p* < 0.001]. Table 3 describes the details.

**Table 3:** Comparison of prevalence of HIV infection among fisherfolk who sought voluntary counselling and testing of HIV (VCT) between September and October 2017 in landing islands of Muleba and Buchosa Districts

Site/ Island	Category	Total tested	HIV+ (%)	OR	95%CI	*p*-value
		*N* = 1744	*n* = 158			
District	Muleba	1394	131 (9.4)	1		
	Buchosa	350	27 (7.7)	1.25	0.812–1.925	> 0.05
Buchosa	Male	223	13 (5.8)	1		
	Female	127	14 (11)	0.499	0.227–1.099	> 0.05
Muleba	Male	806	50 (6.2)	1		
	Female	588	81 (13.8)	0.414	0.286–0.600	< 0.001
Chakazimbwe	Male	70	07 (10)	1		
	Female	108	10 (9.3)	1.088	0.394–3.009	> 0.05
Goziba	Male	281	14 (5)	1		
	Female	141	17 (12.1)	0.382	0.182–0.800	< 0.01
Ihumbo	Male	84	4 (4.8)	1		
	Female	78	4 (5.1)	0.925	0.223–3.833	> 0.05
Iroba	Male	61	2 (3.3)	1		
	Female	59	8 (13.6)	0.216	0.043–1.064	> 0.05
Rubiri	Male	99	2 (2)	1		
	Female	32	4 (12.5)	0.144	0.025–0.829	< 0.05
Makibwa	Male	82	6 (7.3)	1		
	Female	51	14 (27.5)	0.209	0.074–0.587	< 0.001
Mulumo	Male	65	6 (9.2)	1		
	Female	84	11 (13)	0.674	0.236–1.933	> 0.05
Nyabulo	Male	64	9 (14.1)	1		
	Female	35	13 (37.1)	0.277	0.104–0.740	< 0.05
Kerebe	Male	123	6 (2.7)	1		
	Female	64	5 (7.8)	0.605	0.177–2.064	> 0.05
Nyamangu	Male	100	7 (7)	1		
	Female	63	9 (14.35)	0.452	0.159–1.282	> 0.05

### Levels of knowledge and attitude on HIV/AIDS

Only 17.7% of the study participants had an inappropriate level of knowledge about HIV and the rest had adequate knowledge. The measurement of HIV/AIDS knowledge was reliable [Cronbach’s alpha = 0.799]. Attitude on HIV/AIDS was also assessed in this study, about 74% of the respondents had positive attitude towards HIV and AIDS.

### Multivariate logistic regression of factors associated with HIV infection among fisherfolk during the community survey

Factors associated with HIV infection among fisherfolk during the bivariate analysis were: older age, marital status, education and occupation. Others were being not circumcised, no VCT for past 1 year, using alcohol, having multiple sexual partners and working in Buchosa District (Table 4). Upon multivariate logistic regression of the above factors; alcohol users [aOR = 2.26; 95%CI 1.228–4.147], lack of formal education [aOR = 3.37; 95%CI 1.637–6.921; *p* < 0.01] and increasing age [aOR = 1.061; 95%CI 1.029–1.093; *p* < 0.0001] came up as final predictors for increased likelihood of HIV acquisition (Table 5).

**Table 4:** Bivariate analysis of factors associated with HIV infection among fisherfolk in landing sites located in Muleba and Buchosa Districts, north-western Tanzania, October 2017 (*N* = 447)

Category	Variable	Total tested	HIV {+}	Crude OR	95% CI	*p*-value
		*N* = 447	*N* = 63			
Age	≤20	28	1	1		
	21–35	256	32	0.25	0.03–1.97	> 0.05
	≥36	163	30	0.16	0.02–1.25	> 0.05
Location	Muleba	247	27	1		
	Buchosa	200	36	1.79	1.04–3.06	< 0.05
Mean rank age	Age (years)	–	277.25	–	–	< 0.0001 ^a^
Sex	Male	266	32	1		0.16
	Female	181	31	1.51	0.89–2.58	
Marital status	Married	254	24	1		
	Single	101	12	1.29	0.62–2.69	> 0.05
	Widowed/Divorced/	92	27	2.81	1.54–5.13	< 0.001
	Cohabiting					
Education	No formal education	54	19	1		
	Primary+	393	44	4.31	2.27–8.17	< 0.0001
Fisherfolk occupation category	Fishing+farming	91	05			
	Fish business	109	17	3.18	1.12–8.98	< 0.05
	Employed ^b^	67	16	5.40	1.87–15.61	< 0.001
	Fishing	180	25	2.77	1.03–7.51	< 0.05
Religion	Christian	376	54	1		
	Muslim	71	09	1.16	0.54–2.46	0.7
Residence	Permanent	117	13	1		
	Temporary	330	50	1.42	0.75–2.74	0.36
Knowledge	Appropriate	368	50	1		
	Inappropriate	79	13	1.25	0.64–2.44	0.5
HIV Attitude	Positive	331	53	1		
	Negative	116	10	0.49	0.24–1.015	0.06
Condom use	Consistent	234	27	1		
	Inconsistent	213	36	1.55	0.91–2.67	0.10
Circumcision (Only men)	Yes	236	24	1		
	No	30	8	3.21	1.29–8.00	< 0.05
VCT in 1 year	Yes	343	40	1		
	No	104	23	2.15	1.23–3.79	< 0.01
Multiple partners	No	289	31			
	Yes	158	32	1.96	1.15–3.36	< 0.05
Alcohol use	No	275	27	1		
	Yes	172	36	2.43	1.42–4.18	< 0.001

^a^ Mann Whitney test; ^b^ Employed in fishing related business (fish processing, cooking and store keeping)

**Table 5:** Adjusted factors associated with HIV infection among fisherfolk in landing sites located in Muleba and Buchosa Districts, north-western Tanzania, October 2017

Variable	aOR	95% CI		p-value
		Lower	Upper	
Alcohol intake				
Alcohol non-users	1			
Alcohol users	2.26	1.228	4.147	0.009
Place of residence				
Located in Muleba	1			
Located in Buchosa	0.75	0.4	1.401	0.37
Number of sexual partners				
No multiple sexual partners	1			
Multiple sexual partners	0.636	0.349	1.158	0.139
Level of education				
Primary education+	1			
No formal education	3.37	1.637	6.921	0.001
Marital status				
Single	1			
Married	0.563	0.239	1.329	0.19
Cohabit/widow/divorced	0.332	0.167	0.66	0.002
VCT use				
Uptake of VCT in 1 year	1			
No uptake of VCT	0.748	0.382	1.467	0.399
Age of participants				
Age	1.061	1.029	1.093	< 0.001

### Uptake of HIV/AIDS services

The present study evaluated the magnitude of uptake of key HIV/AIDS services, approximately one-third (38%) of male fisherfolk had never tested for HIV within past year before the survey in landing sites located in Buchosa as compared to 16.8% of their counterparts in Muleba. The highest uptake of VCT was among women in Muleba with 90.1% (73/81) whereas in Buchosa only 70% (70/100) of women had ever tested. Only half of all study respondents practiced consistent use of condom and 35.7% (5/14) of respondents found already living with HIV infection and knew their status were not on antiretroviral drugs (ARVs). Moreover, uptake of male circumcision was satisfactory with a coverage of 89% (Table 6).

**Table 6:** Uptake of HIV/AIDS services among fisherfolk in landing sites located in Muleba and Buchosa Districts, north-western Tanzania, October 2017

Variables	Buchosa		Muleba		Total (%)
	Male (%)	Female (%)	Male (%)	Female (%)	
HIV testing for past 12 months					
Yes	62 (62)	70 (70)	138(83)	73 (90.1)	343 (76.7)
No	38 (38)	30 (30)	28 (17)	8(9.9)	104 (23.3)
Condom use last 12 months					
Consistent	42 (42)	49 (48)	94(55.5)	54 (63.5)	239 (52.4)
Inconsistent	58 (58)	53 (52)	75 (44.5)	31 (36.5)	217 (47.6)
HIV infected on antiretroviral therapy					
Yes	0 (0)	1 (50)	4 (57.1)	4 (80)	9 (64.3)
No	0 (0)	1 (50)	3 (42.9)	1 (20)	5 (35.7)
Male circumcision					
Yes	88 (88)	–	151(89.3)	–	239 (88.8)
No	12 (12)	–	18 (10.7)	–	30 (11.2)

## Discussion

This study was able to evaluate the magnitude of HIV infection and uptake of HIV/AIDS services among fisherfolk in Lake Victoria, one of the hard to reach populations. From the community survey, the prevalence of HIV was higher than those who voluntarily requested and tested for HIV. Majority of the people in the community had sufficient knowledge on HIV and a positive attitude towards HIV and AIDS. HIV testing was more common among women. About half of the population used condoms during sexual activities and more than half of the known HIV positives were already on ARVs, however, leaving a third of them not on treatment. Male circumcision was found to be high in this population. Factors that attributed to higher odds of HIV infection included; older age, lack of formal education and using alcohol. Others were one’s type of occupation and marital status.

The prevalence of HIV among fisherfolk who participated in this study was found to be higher than the estimates made among people living in neighbouring regions [20]. However, other studies have also shown that the prevalence among fisherfolk being higher than the general population [10, 13]. In this study, the HIV prevalence was higher during the community survey than from the VCT data. This may highlight the need to have interventions in this population to target the population with the highest risk to be tested. To be able to reach the 90–90-90 goals, scale up of HIV self-testing and linkage to care and treatment to this key and vulnerable population is highly recommended [24, 25].

Majority of the people in the community had sufficient knowledge on HIV, had a positive attitude towards HIV and AIDS and male circumcision was found to be common. This is different from other studies carried out in Asia which found that fisherfolk had lower knowledge on HIV and had a negative attitude towards it [26, 27]. With improved knowledge on HIV and having a positive attitude, may translate into willingness to test one’s status and adhering to medications [28].

Tanzania adopted the test and treat approach in 2016. This approach plays a role in preventing occurrence of compromised immunity and opportunistic infections to the affected individuals. This is important also as with a reduced viral load, an individual is less infectious. It is therefore alarming to note that more than a half of the people who were HIV positive and knew their status were not on ARV. The need to move constantly seeking for fish among fisherfolk [15] may contribute to making access to ARVs and adherence a challenge. The strategy of delivering ARVs from care and treatment clinics to stable client’s residence implemented by the country HIV/AIDS control program may be also be used to improve adherence among HIV infected fisherfolk in islands through mobile clinics. Community health workers may be also used to support delivery of ARVs to stable clients located in hard to reach areas as it has been proved effective in Tanzanian settings [29].

Factors that attributed to HIV infection included; older age, marital status, education and one’s type of occupation. The prevalence of HIV among older age is different from the national average whereby it is most common among those of a younger age. This difference can be because the population among fisherfolk is composed of mainly adult population. The prevalence of HIV being higher among women, those widowed/separated and those with primary education is similar to the estimates at the national level [30]. Those who drink alcohol were more likely to be infected similar to what is known in the general population [31]. Living in an isolated island and idling during the day could have driven the alcohol drinkers fisherfolk to more risky sexual activities [15].

This is the first study to provide an extensive baseline on the prevalence of HIV/AIDS among fishfolk communities among the selected hard to reach islands in Lake Victoria in Northern Tanzania. Such that the recent upsurge of HIV infection in Mwanza and Kagera Regions to some extent could be due to bridging transmission from fishing communities to low risk surrounding areas [20, 32]. The current study therefore provides avenue for further studies among the vulnerable and disadvantaged group, the fishfolk.

### Strength and limitations

The study explored the prevalence of HIV in testing registry and also at the community level and hence brings a true picture of the status. However, due to migration nature of fisherfolk [15], the magnitude of the disease could be underestimated as the current study used a snap shot involving few islands. The true reflection should have involved periods with high and low fish yield as the high yield seasons are known to be with high influx of fisherfolk and the opposite when the produce is low. This study may therefore represent data for the study period and the studied islands.

## Conclusion

The prevalence of HIV among fisherfolk in the studied landing sites is higher than the general population in respective Districts, particularly when tested at the community level. While the population has a sufficient knowledge on HIV and a positive attitude towards it, the uptake of HIV/AIDS services is still not at the recommended volume. We recommend establishment of testing services that consider the mobile nature of the population such as the use of mobile clinics and self HIV testing. The population at highest risk such as widows and women who are separated should be given a top priority during planning for interventions by local health authorities.

## Additional file

Additional file 1: Questionnaire. (DOCX 22 kb)

## Abbreviations

ART: Antiretroviral treatment
VCT: Voluntary counselling and testing
