# Supplementary material for: Prevalence of HIV infection and uptake of HIV/AIDS services among fisherfolk in landing Islands of Lake Victoria, north western Tanzania
Source: BMC Health Serv Res. 2018 Dec 18;18:980. doi: 10.1186/s12913-018-3784-4 (PMC6299499; doi:10.1186/s12913-018-3784-4)
Supplement: Supplementary file 1 — Questionnaire. (DOCX 22 kb) [file 12913_2018_3784_MOESM1_ESM.docx]

**Appendix I:** **Questionnaire**

**Study title:** Prevalence of HIV infection and uptake of HIV/AIDS services among fisherfolk in landing Islands of Lake Victoria, north western Tanzania

District.................................................Division.........................................................................

Ward......................................................Island...........................................................................

GPS...........................................................................................................................................

| **S/N** | **Area** | **Question** | **Response** |
| --- | --- | --- | --- |
| 1 | **Demographic characteristics** | 1. Age | ................................... |
|  |  | 1. Sex | 1. Male 2. Female |
|  |  | 1. Marital status | 1. Married 2. Single 3. Widowed 4. Divorced 5. Cohabiting 6. Others |
|  |  | 1. Education level | 1. Never attend formal education 2. Primary education 3. Secondary education 4. College education 5. University education |
|  |  | 1. Occupation | 1. Fisherman 2. Fish buyer 3. Business man 4. Business lady 5. Employed 6. Local peasant 7. Others.................... |
|  |  | 1. Religion | 1. Christian 2. Muslim 3. Others...................... |
|  |  | 1. Residence | 1. Permanent resident in the island 2. Comes business purposes 3. Others........................... |
|  |  | 1. Alcohol use | 1. Yes 2. No |
| **2** | **Knowledge on HIV/AIDS** | 1.Have you ever heard of HIV and AIDS | 1. Yes 2. No |
|  |  | 2. Do you know the difference between HIV and AIDS | 1. Yes 2. No 3. Not sure |
|  |  | 3. If yes, What is the difference between HIV and AIDS | .................................................... |
|  |  | 4. How can you know if someone is HIV positive | 1. By looking at him or her  2. By blood test  3. If he/she is thin |
|  |  | 5. Can someone get HIV through the following? Circle 1 for “Yes” 2 for “No” and 3 for not sure. | 1. Mosquito bite  2. Sharing utensils  3. Receiving infected blood  4. Sharing injection needles  5.shaking hands with HIV infected individual  6.Hugging with someone with HIV infection  7.Kissing with HIV positive individual  8.Practice unsafe sex with HIV positive individual  9.Being coughed or sneezed by someone with HIV infection  10. Eating food prepared by HIV positive individual  11. Being beaten by some with HIV infection  12. Sharing toilets with HIV positive  13.Sharing smoke with someone with HIV infection |
|  |  | 6. Can you get infected from HIV from any of the following( mark Yes, No or Not sure | 1. Blood  2. Sweat  3. Tears  4. Semen  5. Saliva  6. Urine |
|  |  | 7. Can AIDS be cured? | 1. Yes  2. No  3. Not sure |
|  |  | 8. Is there a vaccine to prevent HIV/AIDS | 1. Yes  2. No  3. Not sure |
|  |  | 9. Can a person prevent him or herself from HIV infection | 1. Getting safe blood  2. Using fresh injections  3. Abstain sex  4. Being faithful  5. Practice safe sex |
|  |  | 10. Would you be willing to have HIV test | 1. Yes  2. No  3. Not sure |
|  |  | 11. Do you think that using condom is good protection against HIV during sexual intercourse | 1. Yes  2.No  3. Not sure |
| **3** | **Attitude on HIV/AIDS** | Please give your opinion on the following statements:  Please indicate use the appropriate number for the following; 1. Agree 2. Disagree 3. May be | a) People with HIV should blame themselves  b) HIV has been sent by GOD to punish people with their sins  c) Some people cannot contract HIV  d) HIV is only for prostates  e) Everyone has equal chance to contract HIV  f) Even if someone practice sexual intercourse once, he/she may get HIV |
|  |  | Please give your opinion on the following statements:  Please indicate the appropriate number for the following; 1. Agree 2. Disagree 3. May be | 1. Heathy looking person cannot have HIV 2. Condoms are a sure way to prevent HIV 3. HIV is a death sentence 4. Sex is the only one to spread HIV 5. HIV always lead to AIDS 6. Using drug abuse protect you from HIV 7. Drinking alcohol protects you from HIV 8. Having sex with a virgin protects you from HIV |
|  |  | If one of your close friends tested HIV positive , would you still associate with them | 1. Yes 2. No 3. Not sure |
| **4** | **HIV practice/ uptake of services** | Have you ever happened to have sexual partner? | 1. Yes 2. 2. No 3. No response |
|  |  | If Yes, how many sexual partners do you currently have? | 1. One 2. Two 3. Three- five 4. More than Five 5. No response |
|  |  | Do you know a condom? | 1. Yes 2. No 3. Not sure |
|  |  | When you are in this working environment, for the past one year, how often do you use condom during sex? | 1. Always 2. Sometimes 3. Never |
|  |  | Have had a chance to meet the team of experts doing male circumcision | 1. Yes 2. No |
|  |  | Did get a chance to be circumcised? | 1. Yes 2. No 3. Not applicable (females) |
|  |  | For the past one years have you ever tested for HIV? | 1. Yes 2. No 3. Not applicable |
|  |  | Are you on ART | 1. Yes 2. No |
|  |  | If on ART, how often do you miss your drugs (dozes) | 1. Very often 2. Sometimes 3. Never happened |
| **5** | **Stigma and discrimination** | If you have HIV or AIDS, would you tell anyone | 1. Yes 2. No 3. Not sure |
|  |  | If yes, who would you tell | 1. My parents 2. My partner 3. Friends 4. Close relatives 5. Others |
|  |  | If you discover you have HIV, would you consider suicide? | 1. Yes 2. No 3. Not sure |
| **6** | **HIV Testing** | Would you wish to know your HIV status? | 1. Yes (pretest counselling) 2. No 3. I’m HIV positive (proof?) |
|  |  | HIV screening results | 1. Positive (posttest counselling and referral) 2. Negative 3. Undetermined |
